# Father's Health Status and Inequalities in Physical and Mental Health of U.S. Children: A Population-Based Study

**DOI:** 10.1089/heq.2019.0051

**Published:** 2019-10-09

**Authors:** Romuladus E. Azuine, Gopal K. Singh

**Affiliations:** ^1^ Division of Research, Office of Epidemiology and Research, Maternal and Child Health Bureau, Health Resources and Services Administration, U.S. Department of Health and Human Services, Rockville, Maryland.; ^2^ Office of Health Equity, Health Resources and Services Administration, U.S. Department of Health and Human Services, Rockville, Maryland.

**Keywords:** father's health, child health, physical health, mental health, child health outcomes, intergenerational health

## Abstract

**Introduction:** Father-and-child-health risk relationship is poorly studied and understood. We examine the impact of father's physical and mental health status and sociodemographic characteristics on the physical and mental outcomes of U.S. children 0–17 years of age.

**Methods:** The 2011–2012 National Survey of Children's Health (*N*=75,879) was analyzed to estimate prevalence and odds of poor physical and mental health among children according to father's physical and mental health status and sociodemographic characteristics.

**Results:** Overall, 3.2% of U.S. children had poor physical health; and 6.0% of U.S. children had emotional or behavioral problems. The adjusted odds of having poor overall health was 3.1 times higher among children of fathers with poor overall health. Children of fathers with poor mental health had 2.6 times higher adjusted odds of having poor mental health.

**Discussion:** Results underscore the significant role of fathers in the physical and mental well-being of children. Engaging fathers in child health may provide a potential opportunity to reduce mental and emotional health problems among children.

## Introduction

Reducing physical, mental, and emotional health problems among U.S. children is a key priority agenda of policy makers, public health experts, and health care professionals to ensure the optimum health and development of children. Child health improvement is a national priority, given that almost one-quarter of U.S. children suffer from at least one chronic health condition, and almost 16% are in poor overall health.^1^ With one in five U.S. children or youth suffering from a mental health or learning disorder^2,3^ and one out of seven 2-to-8-year olds diagnosed with a mental, behavioral, or developmental disorder,^4^ mental health disorders are the most common health issues faced by school-aged children in the United States.^2^ The increasing prevalence of mental health disorders among children and studies showing that 80% of chronic mental disorders begin in childhood necessitate an urgent need to identify the predisposing risk factors and signs of these conditions early in life.^3^ Understanding the multifactorial risk factors for physical, mental, and emotional disorders among children is important; early identification of physical or mental health disorders ensures early initiation of prevention and intervention strategies that could mitigate pathological deterioration and ameliorate consequential disability across generations.

Studies on social determinants of health emphasize the family or home environment as an important place to identify potential determinants as well as risk factors for physical and mental health disorders among children.^5^ In line with this, Healthy People 2020 prioritizes the creation of social and physical environments that promote good health for all and promote quality of life, healthy development, and healthy behaviors across all life stages as an overarching national goal.^6^ The home environment is the foundation for intergenerational continuity of positive or negative behaviors from parents to their children. Intergenerational transmission of the deleterious effects of adverse childhood experiences, economic adversity, and poor health outcomes from parents to offspring has been documented.^7^ Studies have shown that a positive home-rearing environment exerts a positive influence on children's behavioral problems,^8^ and that home parenting policies are associated with physical activity and healthy food intake among offspring.^9^

The majority of intergenerational health studies on the parent-child mental health nexus have focused on parental socioeconomic status or environmental factors, mothers, and less specifically on fathers.^10,11^ However, a growing body of research has documented the importance of fathers in the intergenerational transmission of positive and negative behaviors between fathers and their children, thus indicating unique relationships between father-child physical and mental health relationships.^12,13^ Associations between father involvement and positive cognitive, developmental, and sociobehavioral child outcomes such as improved weight gain in pre-term infants, improved breastfeeding rates, higher receptive language skills, and higher academic achievement have been reported.^13,14^ A father in better mental health buffers the influence of a mother's poorer mental health on a child's behavioral and emotional problems.^15^ The risk of a child exhibiting high externalizing behavioral problems is elevated if both parents reported poorer mental health, but is less elevated if only the mother reported poorer mental health in comparison to the father.^15^ Early-onset cannabis use by a father is indirectly associated with his child's onset of cannabis use through father's lifetime cannabis disorder.^16^ Paternal depression also exerts unique and long-term effects on children's risk for mood disorders, with these effects persisting into adulthood.^9^ Existing studies examining paternal-child influences are limited by small sample sizes, lack of large, national or population-based analysis, and nonexploration of the interplay between overall health and mental health indicators between fathers and children.

With an estimated 24 million children living in father-absent homes in the United States,^17,18^ increasing the involvement of fathers and father-figures in child health and development has become an issue of increasing interest to health care practitioners, and social and public policy makers since the early 1990s, given the benefits of father involvement in child health and development outcomes.^19,20^ A better understanding of how fathers' physical and mental health shape the mental and emotional health of their next generation may lead to new opportunities for public health prevention interventions engaging fathers with potential for achieving equity in health and development outcomes among children. This study (1) examines the association between father's physical and mental health and their child's physical, mental, and emotional status; (2) determines whether the associations between father's physical and mental health and their child's physical and mental health vary by child's age and gender; and (3) identifies potential protective factors in the association between father's mental health and that of their children. We hypothesize a positive intergenerational association between fathers' physical and mental health and those of their children.

## Methods

Data for this study were from the 2011–2012 National Survey of Children's Health (NSCH). With funding and direction from the Health Resources and Services Administration's Maternal and Child Health Bureau, the NSCH is a nationally representative survey designed to assess the physical and emotional health of 95,677 children 0–17 years of age, as well as factors that may relate to child well-being, including medical homes, family interactions, parental health, school and after-school experiences, and neighborhood characteristics.^21^ The 2011–2012 NSCH is a cross-sectional telephone survey of U.S. households with at least one resident child younger than 18 years at the time of the interview. One child was randomly selected to be the subject of the detailed interview if more than one child lived in the household. In households with one child, that child was selected to be the subject of the detailed interview. One parent or guardian with knowledge of the health and health care of the sampled child in the household completed the survey. The survey was conducted in all 50 states and DC between February 2011 and June 2012.^21^ All survey data were based on parental/guardian reports. The interview completion rate for the 2011–2012 NSCH, a measure of the response rate indicating the percentage of completed interviews among known households with a child younger than 18 years, was 54.1% for the landline sample and 41.2% for the cell phone sample.^21^ Detailed information about the NSCH can be found elsewhere.^21^ The NCHS Research Ethics Review Board approved all data collection procedures for the survey.

### Study variables

Our study had two dependent variables: (1) child's overall physical health status and (2) child's mental health status. Our study's main independent variables were (1) father's overall physical health status and (2) father's mental health status. In selecting our dependent and independent variables, we drew upon the World Health Organization's holistic definition of health as “the state of complete physical, mental, and social well-being and not merely the absence of disease or infirmity.”^5^ In our study, father refers to biological, step, foster, or adoptive fathers, who lived with their children at the time of survey interview.

Children's overall physical health was defined based on the survey response by parents who were asked, “in general, how would you describe [child name]'s health? Would you say [his/her] health is excellent, very good, good, fair, or poor?” Children's overall mental health consisted of responses to survey questions on the prevalence of one or more chronic conditions with regard to depression, anxiety, and conduct disorders, including oppositional defiant disorders. For each condition, respondents were asked whether they have ever been told by a health care professional that the child has the condition, and whether the child currently has the conditions. We combined these responses into a composite emotional/behavioral problem (EBP) variable consistent with previously published and accepted categorizations.^22^ For each of the conditions, the respondents were asked whether a doctor or health care provider had ever told them that the child had each of the following conditions: *depression, anxiety, or behavioral or conduct problems, such as oppositional defiant disorder or conduct disorder. They were also asked if the child currently had any of the conditions.* This composite EBP global mental health indicator was defined in the survey for children 2–17 years of age as previously described.^1^

Father's overall physical health was defined based on responses to the following survey question: “in general, what is the status of [child name]'s [father's/your] health? Respondents rated fathers' health status as excellent/very good, good, or fair/poor. We dichotomized this variable into “good overall health status” for excellent/very good/good responses or “poor health status” for fair/poor responses. Similarly, father's mental health was defined as a dichotomous variable with their mental and emotional health being either excellent/very good/good or fair/poor. To derive this response, fathers in the survey were asked, “in general, what is the status of [child name]'s [father's/your] mental and emotional health?” For both questions on father's health, when the respondent was the target child's father (biological, step, foster, and adoptive), he rated his own physical health. However, respondents who were not the target child's father (e.g., mother or other relative) gave a rating of the father's physical health. All responses were combined regardless of whether the person answering was the father himself or another respondent, but excluded ∼20% of children in the survey who were not currently living with a father in the household.

Using a life-course health development^23^ and social-determinants-of-health frameworks,^24^ and guided by past research, we considered the following socioeconomic and demographic variables as covariates in the study: child's age, sex, race/ethnicity; household composition; metropolitan or nonmetropolitan residence; primary language spoken at home; highest parental education in years of school completed; and household poverty status measured as a ratio of family income to the federal poverty level.^22^ These covariates are measured as shown in Table 1.

**Table 1.** Observed (Unadjusted) Prevalence of Overall Health (*N*=75,879) and Mental Health Status (*N*=67,524) Among U.S. Children 0–17 Years of Age by Father's Overall and Mental Health Status and Sociodemographic Characteristics: The 2011–2012 National Survey of Children's Health

Covariate	Study sample all children (*N*=75,879)		Child's overall health status as fair or poor^a^		Child has emotional/behavioral health problem^b^	
	%	SE	%	SE	%	SE
United States	100%		3.16	0.14	6.04	0.18
Father's overall health status						
Fair or poor	8.62	0.25	10.66	0.99	9.49	0.90
Excellent, very good, or good	91.38	0.25	1.81	0.13	4.35	0.19
Father's mental health status						
Fair or poor	4.63	0.17	9.83	1.18	14.53	1.37
Excellent, very good, or good	95.37	0.17	2.21	0.14	4.32	0.19
Child's age, years						
0–5	32.76	0.34	2.58	0.22	2.03	0.23
6–11	33.19	0.35	3.37	0.27	6.17	0.30
12–17	34.06	0.35	3.50	0.23	8.46	0.35
Child's sex						
Male	51.15	0.37	3.38	0.21	7.12	0.27
Female	48.85	0.37	2.92	0.19	4.90	0.24
Race/ethnicity						
Non-Hispanic white	51.13	0.36	1.70	0.10	6.79	0.24
Non-Hispanic black	13.22	0.24	4.26	0.40	6.10	0.47
Hispanic	23.04	0.37	6.12	0.49	4.79	0.46
Non-Hispanic mixed race	4.78	0.13	2.58	0.43	8.11	0.79
Other	7.82	0.21	2.46	0.32	3.33	0.44
Household composition						
Two-parent biological	64.9	0.36	2.42	0.16	3.83	0.18
Two parent	8.66	0.22	3.77	0.57	9.99	0.82
Single	18.85	0.29	5.34	0.42	9.74	0.50
Other family type	7.59	0.21	3.29	0.36	9.82	0.85
Place of residence						
Metropolitan	82.13	0.24	3.18	0.16	6.04	0.21
Nonmetropolitan	17.87	0.24	3.02	0.24	6.05	0.33
Primary language spoken at home						
English	84.39	0.33	2.31	0.11	6.61	0.20
Any other language	15.61	0.33	7.71	0.66	2.83	0.48
Household/parental education level, years						
<12	11.44	0.28	9.15	0.80	6.83	0.69
12	19.98	0.31	4.25	0.36	7.11	0.43
12+	68.58	0.37	1.76	0.11	5.73	0.21
Household poverty status (ratio of family income to poverty threshold)						
Below 100%	22.45	0.33	7.05	0.45	9.05	0.50
100–199%	21.64	0.32	3.58	0.34	6.66	0.41
200–399%	28.13	0.33	2.03	0.23	5.18	0.36
At or above 400%	27.78	0.31	0.81	0.08	4.06	0.24

^a^ Children 0–17 years of age.

^b^ Children 2–17 years of age. The chi-square test for the overall association between each covariate and the two outcomes was statistically significant at *p*<0.01 level, except for the association of child's place of residence with child's overall health status and mental health status and of child's sex with child's overall health status.

SE, standard error.

### Statistical analysis

We modeled the odds of child's poor overall health, child's emotional and behavioral disorders, among 75,879 children as a function of father's overall health status and father's mental health status and pertinent sociodemographic covariates described above. Children with missing data on father's overall health and mental health status were excluded from all analyses. Prevalence (%) estimates of both outcome variables were computed for all covariates and for all children in the survey with the outcomes of interest. The χ^2^ statistic was used to test the overall association between each covariate and the two outcomes. Weighted logistic regression was used to examine the association between father's overall health and mental health and child's overall health and mental health status, after adjusting for covariates. To account for the complex sample design of the NSCH, SUDAAN software was used to conduct all statistical analyses.^25^

## Results

Table 1 presents the observed prevalence of children's overall health status and emotional and behavioral health disorders according to father's overall health and mental health status. About 3.2% U.S. children were reported to have poor overall health status, and 6.0% of children had emotional or behavioral health problems. Prevalence of poor overall health status was higher among children whose fathers reported poor health status. About 10.7% of U.S. children whose fathers had poor health also had poor overall health status, compared to 1.8% of children whose fathers had good overall health. About 9.8% of children whose fathers had poor mental health were reported to have poor overall health status, compared to 2.2% of children whose fathers had good mental health status. Father's mental health status was associated with children's mental health status. About 14.5% of children whose fathers had poor mental health status had emotional or behavioral disorders compared to 4.3% of children whose fathers had good mental health status.

Table 2 presents the unadjusted and adjusted odds of poor overall health and mental health among U.S. children by father's overall health and mental health status and sociodemographic characteristics. Before statistical adjustment, children of fathers with poor overall health status had 6.5 and 2.3 times higher odds, respectively, of having poor overall health and poor mental health, compared to children whose fathers had good overall health. Before adjustment, children of fathers with poor mental health had 4.8 and 3.8 times higher odds, respectively, of having poor overall health and poor mental health compared to children of fathers with good mental health. Before adjustments, Hispanic and non-Hispanic black children had 3.8 and 2.6 times higher unadjusted odds, respectively, of having overall poor health compared with non-Hispanic white children. Children below the poverty level had 9.3 times higher odds of being in poor overall health compared to their counterparts from affluent households.

**Table 2.** Unadjusted and Adjusted Odds of Fair/Poor Overall Health and Mental Health Among U.S. Children 0–17 Years of Age by Father's Overall Health and Mental Health Status and Sociodemographic Characteristics: The 2011–2012 National Survey of Children's Health

Covariate	Child in fair/poor overall health unadjusted odds		Child in fair/poor overall health adjusted odds^a^		Child in fair/poor mental health unadjusted odds		Child in fair/poor mental health adjusted odds^a^	
	OR	95% CI	AOR	95% CI	AOR	95% CI	AOR	95% CI
Father's overall health status								
Fair or poor	6.49	5.06–8.32	3.14	2.33–4.23	2.31	1.84–2.89	1.48	1.11–1.99
Excellent, very good, or good	1.00	Reference	1.00	Reference	1.00	Reference	1.00	Reference
Father's mental health status								
Fair or poor	4.81	3.59–6.45	1.94	1.36–2.75	3.77	2.98–4.76	2.64	2.00–3.49
Excellent, very good, or good	1.00	Reference	1.00	Reference	1.00	Reference	1.00	Reference
Child's age, years								
0–5	1.00	Reference	1.00	Reference	1.00	Reference	1.00	Reference
6–11	1.31	1.03–1.67	1.19	0.87–1.62	3.17	2.47–4.06	2.80	2.00–3.91
12–17	1.37	1.10–1.71	1.42	1.05–1.91	4.45	3.49–5.67	3.79	2.74–5.26
Child's sex								
Male	1.16	0.97–1.39	1.18	0.93–1.49	1.49	1.31–1.69	1.44	1.21–1.71
Female	1.00	Reference	1.00	Reference	1.00	Reference	1.00	Reference
Race/ethnicity								
Non-Hispanic white	1.00	Reference	1.00	Reference	1.00	Reference	1.00	Reference
Non-Hispanic black	2.58	2.06–3.24	1.60	1.11–2.30	0.89	0.75–1.07	0.54	0.40–0.73
Hispanic	3.77	3.07–4.64	1.46	0.98–2.17	0.69	0.56–0.86	0.78	0.56–1.07
Non-Hispanic mixed race	1.53	1.07–2.19	1.27	0.77–2.09	1.21	0.97–1.51	0.71	0.51–0.99
Other	1.46	1.09–1.95	1.08	0.71–1.64	0.47	0.36–0.63	0.56	0.39–0.80
Household composition								
Two-parent biological	1.00	Reference	1.00	Reference	1.00	Reference	1.00	Reference
Two-parent stepfamily	1.58	1.13–2.20	1.39	0.94–2.05	2.79	2.28–3.42	2.00	1.58–2.52
Single mother	2.27	1.85–2.80			2.71	2.34–3.14		
Other family type	1.37	1.06–1.77	0.83	0.54–1.29	2.74	2.22–3.38	1.53	1.03–2.28
Place of residence								
Metropolitan	1.05	0.87–1.28	1.06	0.85–1.33	1.00	0.87–1.14	1.35	1.13–1.61
Nonmetropolitan	1.00	Reference	1.00	Reference	1.00	Reference	1.00	Reference
Primary language spoken at home								
English	1.00	Reference	1.00	Reference	1.00	Reference	1.00	Reference
Any other language	3.53	2.87–4.34	1.82	1.21–2.73	0.41	0.29–0.58	0.36	0.21–0.61
Household/parental education level, years								
<12	5.63	4.49–7.07	1.91	1.29–2.84	1.21	0.96–1.51	1.00	0.68–1.46
12	2.48	2.00–3.08	1.46	1.07–1.98	1.26	1.08–1.46	0.80	0.63–1.01
12+	1.00	Reference	1.00	Reference	1.00	Reference	1.00	Reference
Household poverty status (ratio of family income to poverty threshold)								
Below 100%	9.29	7.30–11.82	3.35	2.28–4.93	2.35	1.98–2.78	2.91	2.22–3.83
100–199%	4.55	3.41–6.07	2.23	1.55–3.20	1.69	1.42–2.01	1.86	1.45–2.38
200–399%	2.54	1.88–3.42	2.38	1.65–3.43	1.29–1.07	1.55–1.31	1.04–1.64	
At or above	400%	1.00	Reference	1.00	Reference	1.00	Reference	1.00

^a^ Adjusted by multiple logistic regression for father's overall and mental health status, child's age, sex, race/ethnicity, household language use, household composition, metropolitan/nonmetropolitan residence, parental education, and household poverty status.

AOR, adjusted odds ratio; CI, confidence interval; OR, odds ratio.

After controlling for socioeconomic and demographic characteristics, statistically significant associations between father's overall health and mental health status and children's physical and mental health status persisted (Table 2). Children of fathers with poor overall health status had 3.1 times higher adjusted odds of having poor overall health compared to children whose fathers had good overall health. Similarly, children of fathers with poor physical health status had 1.5 times higher adjusted odds of having correspondingly poor mental health compared to children of fathers with good or excellent mental health status. Children of fathers with poor mental health status had 1.9 times higher adjusted odds of having poor overall health compared to children whose fathers reported good mental health status. Regarding father-child mental health association, we found that children of fathers with poor mental health had 2.6 times higher adjusted odds of having poor mental health status compared to children whose fathers reported good mental health.

Table 3 shows that the association between father's physical and mental health and child's physical and mental health varies significantly by child's age and gender. Father's overall health status seems to have a stronger influence on younger children's than older children's overall health and mental health status. Father's mental health, on the other hand, appeared to have a stronger association with older children's physical and mental health.

**Table 3.** Weighted Prevalence (%) and Unadjusted and Adjusted Odds of Fair/Poor Overall Health and Mental Health Among U.S. Children 0–17 Years of Age by Father's Overall Health and Mental Health Status: The 2011–2012 National Survey of Children's Health

Covariate	Child's overall health status as fair or poor		Child in fair/poor overall health Unadjusted odds		Child in fair/poor overall health Adjusted odds^a^		Child's mental health status as fair or poor		Child in fair/poor mental health Unadjusted odds		Child in fair/poor mental health Adjusted odds^a^	
	%	SE	OR	95% CI	AOR	95% CI	%	SE	OR	95% CI	AOR	95% CI
Father's overall health status												
Males 0–5 years of age												
Fair or poor	9.66	2.18	6.35	3.53–11.41	3.96	2.21–7.11	6.21	2.94	2.97	1.04–8.52	2.33	0.67–8.12
Excellent/very good/good	1.66	0.27	1.00	Reference	1.00	Reference	2.18	0.39	1.00	Reference	1.00	Reference
Males 6–11 years of age												
Fair or poor	8.27	1.67	3.56	2.00–6.33	2.13	0.99–4.58	12.06	2.22	2.18	1.39–3.43	1.41	0.85–2.35
Excellent/very good/good	2.47	0.47	1.00	Reference	1.00	Reference	5.91	0.53	1.00	Reference	1.00	Reference
Males 12–17 years of age												
Fair or poor	9.93	1.88	4.71	2.77–8.00	3.14	2.33–4.23	9.62	1.65	1.52	1.01–2.27	1.05	0.63–1.75
Excellent/very good/good	2.29	0.38	1.00	Reference	1.00	Reference	6.55	0.5	1.00	Reference	1.00	Reference
Females 0–5 years of age												
Fair or poor	12.70	4.01	11.34	5.13–25.06	5.03	1.88–13.48	2.74	1.56	3.93	0.95–16.23	1.91	0.19–19.54
Excellent/very good/good	1.27	0.23	1.00	Reference	1.00	Reference	0.71	0.3	1.00	Reference	1.00	Reference
Females 6–11 years of age												
Fair or poor	11.35	2.36	9.45	5.24–17.02	4.19	2.07–8.48	7.40	2.36	2.69	1.32–5.47	2.69	1.09–6.67
Excellent/very good/good	1.34	0.25		Reference	1.00	Reference	2.89	0.32	1.00	Reference	1.00	Reference
Females 12–17 years of age												
Fair or poor	12.36	2.40	7.73	4.69 12.76	2.97	1.55 5.70	13.41	2.26	2.50	1.63 3.83	1.48	0.84 2.6
Excellent/very good/good	1.79	0.22		Reference	1.00	Reference	5.84	0.56	1.00	Reference	1.00	Reference
Father's mental health status												
Males 0–5 years of age												
Fair or poor	6.46	2.12	3.40	1.62–7.17	1.40	0.68–2.88	4.73	2.6	2.05	0.62–6.71	1.34	0.41–4.41
Excellent/very good/good	1.99	0.29	1.00	Reference	1.00	Reference	2.37	0.43	1.00	Reference	1.00	Reference
Males 6–11 years of age												
Fair or poor	6.20	1.43	2.29	1.27–4.12	1.34	0.62–2.93	20.11	3.54	4.15	2.59–6.65	2.88	1.70–4.89
Excellent/very good/good	2.81	0.47	1.00	Reference	1.00	Reference	5.71	0.52	1.00	Reference	1.00	Reference
Males 12–17 years of age												
Fair or poor	8.36	2.25	3.12	1.64 5.94	1.44	0.62 3.35	19.73	3.08	3.75	2.48 5.67	3.50	2.08 5.89
Excellent/very good/good	2.84	0.4	1.00	Reference	1.00	Reference	6.15	0.47	1.00	Reference	1.00	Reference
Females 0–5 years of age												
Fair or poor	12.53	4.94	8.21	3.12–21.59	2.48	0.75–8.25	3.04	1.39	3.96	1.18–13.26	1.94	0.33–11.23
Excellent/very good/good	1.71	0.34	1.00	Reference	1.00	Reference	0.79	0.31	1.00	Reference	1.00	Reference
Females 6–11 years of age												
Fair or poor	10.07	2.57	6.33	3.31–12.10	1.96	0.90–4.29	5.96	1.58	1.96	1.08–3.58	1.42	0.67–3.01
Excellent/very good/good	1.74	0.29	1.00	Reference	1.00	Reference	3.13	0.37	1.00	Reference	1.00	Reference
Females 12–17 years of age												
Fair or poor	15.37	3.52	8.27	4.61–14.85	3.70	1.77–7.70	20.46	3.65	4.17	2.58–6.73	3.09	1.82–5.26
Excellent/very good/good	2.15	0.27	1.00	Reference	1.00	Reference	5.81	0.54	1.00	Reference	1.00	Reference

^a^ Adjusted for father's overall health and mental health status, race/ethnicity, household language use, household composition, metropolitan/nonmetropolitan residence, parental education, and household poverty status.

## Discussion

The optimum development of all children and their attainment of age-appropriate physical and mental health outcomes are of great interest to researchers, pediatric professionals, and public policy experts in the fields of public health and education. We found that children whose fathers had poor mental health also reported higher emotional and behavioral health problems compared to children whose fathers had good mental health.

Our findings have a number of implications. First, the synchronicity of data on mental health status of fathers and their children presents a vista of opportunity for interventions. The results provide evidence that interventions could equally be effective when implemented among fathers individually or among father-child dyads. With this information, it is potentially possible that fathers, who are traditionally hard to recruit into community-based interventions, could be better persuaded when presented with evidence that their health matters in the health and well-being of their children. Health care practitioners could find these data useful in framing opportunities to discuss father-involvement in the lives of children as part of the anticipatory guidance for families at health care visits. For visits where fathers are involved, these data may be useful in reinforcing the benefits of father involvement in the health and well-being of children. The American Academy of Pediatrics' Bright Futures espouses the need for health care practitioners to promote family support for health care supervision of infants, children, and adolescents.^26^ In many western countries, the role of fathers in child health and development has often been relegated to the background. Unlike mothers, data on the benefits of father involvement in child health have been sparse, further lending credence to the poor engagement of fathers on the health of their children. Social policy makers acknowledge that lack of father engagement in child health policies and programs might present a missed opportunity for optimal child development and strengthening of families as the fundamental unit of positive health behaviors.^27,28^

In our study, we found that the prevalence of EBPs ranged from a low of 4.8% for Hispanic children to highs of 6.1% and 6.8% for non-Hispanic black and non-Hispanic white children, respectively. These results indicate that, if well planned and intentionally implemented, father-involvement interventions for child mental health could equally benefit all children regardless of race/ethnicity. After accounting for socioeconomic differences, we found that children from two-parent step families and other family types with a father present had higher adjusted odds, respectively, of poor mental health compared to children from two-parent biological households. Although the odds of poor mental health persisted for children regardless of household composition, it was slightly reduced across the board indicating the positive associations of father's presence (biological, step or adoptive) on child's emotional and mental health. Although these positive associations are modest, the father absence crisis in America means that the 19.7 million children, or 1 in 4 living without a father in their homes, may never leverage these positive outcomes.^29^ Public policy makers, program planners, child health experts, and community leaders can leverage these findings in the design of strategies aimed at averting the plethora of potential deleterious societal ills and suboptimal health outcomes associated with father's absence.^29^ Moreover, the importance of the household or family is critical, given that most mental health disorders follow a developmental course that typically starts early in life.^30^

### Limitations

Our study has limitations. Children's overall (physical) health and emotional and behavioral disorders examined in our study are based on parental/guardian reports and may not accurately reflect the actual prevalence. Second, since NSCH is a cross-sectional survey, we are unable to draw causal inferences from the data. Furthermore, NSCH is a household-based survey with respondents drawn from households with telephone access. It is possible that some children in transitory homes, migrants, or institutionalized children may have been excluded from the survey, so are also 20% of children who were not currently living with a father in the household and were not included in the survey.

As it is typical of most sample surveys, there is a potential for nonresponse bias in the NSCH, implying that the sample interviewed differed from the targeted child population in a systematic manner.^31^ The response rates in the NSCH tend to be lower in urban areas and low-income and ethnic-minority populations, thus, differential nonresponse bias might affect (most likely underestimate) the impact of father's health, individual socioeconomic status, and race/ethnicity on the physical and mental health of children.^31^ However, the nonresponse adjustment to the sampling weights in the NSCH might have reduced the potential magnitude of these biases.^31^ The increasing use of cell/mobile phone use in recent years, especially among young, minority, renters, and low-income adults, may be an additional source of noncoverage bias.^32,33^ Two-thirds of the total respondents in the 2011–2012 NSCH were from the landline sample and one-third were from the cell/mobile phone sample. The interview completion rate was 54.1% for the landline sample and 41.2% for the cell phone sample. Hence, the nonresponse bias was likely greater in the cell phone sample compared to the landline sample.^31^

There is potential recall bias for respondents answering survey questions several months after the event of interest; in this case, father's overall health and mental health status and children's overall health and emotional and behavioral disorders. Nonetheless, some methodological and design elements in NSCH and our analysis address the aforementioned limitations. For example, NSCH employs nonresponse adjustment to the sampling weights that reduces the magnitude of sample and response biases. Further studies are needed to examine the temporality of events, specifically the reverse effects of children's health on father's health. Since NSCH is a large nationally representative survey, our findings are representative of children 0–17 years of age in the United States.

## Conclusion

In addition to moderating mental and emotional health problems, children with involved, loving fathers are significantly more likely to do well in school, have healthy self-esteem, and exhibit empathy and prosocial behavior compared to children who have uninvolved fathers.^30^ Involved fathers, in addition, provide practical support in raising children and serve as models for their development into adulthood and responsible citizens.^34^ Our study provides important population-based data supporting the need to engage fathers in programs aimed at improving child health and development outcomes. In conclusion, our study further supports the inextricable relationship between the health and well-being of children to their parents' physical, emotional, and social health, social circumstances, and child-rearing practices, which prior studies have examined.^35^ However, in our study, we take the exploration of this inextricable relationship a step further by providing robust data that support the importance of the father in this relationship between the overall and mental health of fathers and their children. Father involvement provides an untapped opportunity for policy makers and program planners to style policies and design programs that foster the health and well-being of children and all families that we can no longer afford to miss.

## Abbreviations Used

AOR: adjusted odds ratio
CI: confidence interval
EBP: emotional/behavioral problem
NSCH: National Survey of Children's Health
OR: odds ratio
SE: standard error

## References

[1] U.S. Department of Health and Human Services, Health Resources and Services Administration, Maternal and Child Health Bureau. The health and well-being of children: a portrait of states and the nation, 2011–2012. 2014. 2016. Available at https://mchb.hrsa.gov/nsch/2011-12/health/ Accessed 52, 2019

[2] Child Mind Institute. 2016 Children's Mental Health Report. 2016 Available at https://childmind.org/ Accessed 52, 2019

[3] Merikangas KR, He JP, Burstein M, et al. Lifetime prevalence of mental disorders in U.S. adolescents: results from the National Comorbidity Survey Replication—Adolescent Supplement (NCS-A). J Am Acad Child Adolesc Psychiatry. 2010;49:980–989 20855043 10.1016/j.jaac.2010.05.017 PMC2946114

[4] Bitsko RH, Holbrook JR, Robinson LR, et al. Health care, family, and community factors associated with mental, behavioral, and developmental disorders in early childhood—United States, 2011–2012. MMWR Morb Mortal Wkly Rep. 2016;65:221–226 26963052 10.15585/mmwr.mm6509a1

[5] World Health Organization. Preamble to the Constitution of the World Health Organization as adopted by the International Health Conference, New York, June 19–22, 1946; signed on July 22, 1946 by the representatives of 61 States (Official Records of the World Health Organization, no. Geneva, Switzerland: World Health Organization). 1948 Available at https://www.who.int/governance/eb/who_constitution_en.pdf Accessed 12, 2016

[6] U.S. Department of Health and Human Services. About healthy people 2020. 2014 Available at https://www.healthypeople.gov/2020/About-Healthy-People Accessed 1124, 2016

[7] American Academy of Pediatrics Council on Community Health. Poverty and child health in the United States. Pediatrics. 2016;13727543009

[8] Chen W, Tanaka E, Watanabe K, et al. The influence of home-rearing environment on children's behavioral problems 3 years' later. Psychiatry Res. 2016;244:185–193 27485757 10.1016/j.psychres.2016.07.043

[9] Reeb BT, Wu EY, Martin MJ, et al. Long-term effects of fathers' depressed mood on youth internalizing symptoms in early adulthood. J Res Adolesc. 2015;25:151–162 25750495 10.1111/jora.12112 PMC4350018

[10] Bond MJ The missing link in MCH: paternal involvement in pregnancy outcomes. Am J Mens Health. 2010;4:285–286 21131334 10.1177/1557988310384842

[11] Case A, Paxson C Parental behavior and child health. Health Aff. 2002;21:164–178 10.1377/hlthaff.21.2.164 11900156

[12] Kroll ME, Carson C, Redshaw M, et al. Early father involvement and subsequent child behaviour at ages 3, 5 and 7 years: prospective analysis of the UK Millennium Cohort Study. PLoS One. 2016;11:e0162339 27654635 10.1371/journal.pone.0162339 PMC5031314

[13] Panter-Brick C, Burgess A, Eggerman M, et al. Practitioner review: engaging fathers—recommendations for a game change in parenting interventions based on a systematic review of the global evidence. J Child Psychol Psychiatry 2014;55:1187–1212 24980187 10.1111/jcpp.12280 PMC4277854

[14] Wilson KR, Prior MR Father involvement and child well-being. J Paediatr Child Health. 2011;47:405–407 20598076 10.1111/j.1440-1754.2010.01770.x

[15] Kahn RS, Brandt D, Whitaker RC Combined effect of mothers' and fathers' mental health symptoms on children's behavioral and emotional well-being. Arch Pediatr Adolesc Med. 2004;158:721–729 15289242 10.1001/archpedi.158.8.721

[16] Henry KL, Augustyn MB Intergenerational continuity in cannabis use: the role of parent's early onset and lifetime disorder on child's early onset. J Adolesc Health. 2017;60:87–92 27838235 10.1016/j.jadohealth.2016.09.005 PMC5333989

[17] U.S. Census Bureau CPS. Living Arrangements of Children under 18 Years/1 and Marital Status of Parents by Age, Sex, Race, and Hispanic Origin/2 and Selected Characteristics of the Child for all Children 2010. Washington, DC: U.S. Census Bureau, 2010

[18] Vespa J, Lewis JM, Kreider RM America's Families and Living: 2012. Washington, DC: U.S. Census Bureau, 2013

[19] Adamsons K, Johnson SK An updated and expanded meta-analysis of nonresident fathering and child well-being. J Fam Psychol. 2013;27:589–599 23978321 10.1037/a0033786

[20] Yogman M, Garfield CF, American Academy of Pediatrics Committee on Psychosocial Aspects of Child and Family Health. Fathers' roles in the care and development of their children: the role of pediatricians. Pediatrics. 2016;138;pii: 10.1542/peds.2016-1128 27296867

[21] U.S. Centers for Disease Control and Prevention, National Center for Health Statistics. State and Local Area Integrated Telephone Survey. 2011–2012 National Survey of Children's Health Frequently Asked Questions. 2013 https://www.cdc.gov/nchs/slaits/index.htm, 2016

[22] Singh GK, Kenney MK, Ghandour RM, et al. Mental health outcomes in US children and adolescents born prematurely or with low birthweight. Depress Res Treat. 2013;2013:570743 24324882 10.1155/2013/570743 PMC3845867

[23] Ben-Shlomo Y, Kuh D A life course approach to chronic disease epidemiology: conceptual models, empirical challenges and interdisciplinary perspectives. Int J Epidemiol. 2002;31:285–293 11980781

[24] Healthy People 2020. Social Determinants of Health. 2014 Available at https://www.healthypeople.gov/2020/topics-objectives/topic/social-determinants-of-health Accessed 52, 2019

[25] Research Triangle Institute, SUDAAN Release 11.0.1, Research Triangle Park, NC, 2013

[26] American Academy of Pediatrics, Promoting Family Support, In Bright Futures. Guidelines for Health Supervision of Infants, Children, and Adolescents. 3rd 2016 Available at https://brightfutures.aap.org/materials-and-tools/guidelines-and-pocket-guide/Pages/default.aspx Accessed 52, 2019

[27] Lu MC, Jones L, Bond MJ, et al. Where is the F in MCH? Father involvement in African American families. Ethn Dis. 2010;20(1 Suppl 2):S2-49-61 20629247

[28] Ramchandani P, Iles J Commentary: getting fathers into parenting programmes—a reflection on Panter-Brick et al. (2014). J Child Psychol Psychiatry. 2014;55:1213–1214 25135767 10.1111/jcpp.12321

[29] National Fatherhood Initiative. Father absence+involvement statistics. 2017 Available at https://www.fatherhood.org/fatherhood-data-statistics Accessed 724, 2019

[30] Garfield CF, Duncan G, Rutsohn J, et al. A longitudinal study of paternal mental health during transition to fatherhood as young adults. Pediatrics. 2014;133:836–843 24733877 10.1542/peds.2013-3262 PMC4006439

[31] Bramlett MD, Blumberg SJ, Zablotsky B, et al. Design and operation of the national survey of children's health, 2011–2012. Vital Health Stat. 2017;1:1–246 28796596

[32] Blumberg SJ, Luke JV Reevaluating the need for concern regarding noncoverage bias in landline surveys. Am J Public Health. 2009;99:1806–1810 19696381 10.2105/AJPH.2008.152835 PMC2741531

[33] Blumberg SJ, Julian VL Wireless Substitution: Early Release of Estimates from the National Health Interview Survey, January–June 2008. Hyattsville, MD: National Center for Health Statistics, 2008

[34] U.S. Department of Health and Human Services, Administration for Children and Families. Responsible Fatherhood. 2016 Available at https://www.acf.hhs.gov/ofa/programs/healthy-marriage/responsible-fatherhood Accessed 1215, 2016

[35] Schor EL Family pediatrics: report of the task force on the family. Pediatrics. 2003;111(6 Pt 2):1541–1571 12777595

